# Professional quality of life and coping strategy of trauma nurses- a cross-sectional study

**DOI:** 10.1007/s00068-025-02975-8

**Published:** 2025-10-28

**Authors:** Ismail Mohamed Eysa, Heba Shehada, Maricar Rulloda-Agoncillo, Nesiya Pallippattu Hassan, Asmaa Mosa AlAtey, Kalpana Singh

**Affiliations:** 1 Hamad Medical Corporation, Doha, Qatar; 2 Nursing Research Department, Hamad Medical Corporation, Doha, Qatar

## Abstract

**Background:**

This study investigates the professional quality of life and coping strategies among trauma nurses in Qatar, aiming to understand the factors contributing to burnout, compassion fatigue, and secondary traumatic stress.

**Study design and methods:**

Utilizing a cross-sectional design, data were collected from 156 trauma nurses at Hamad General Hospital between February and May 2023. The Professional Quality of Life Scale (ProQOL) and Brief-COPE were employed to measure compassion satisfaction, burnout, secondary traumatic stress, and coping strategies. Descriptive statistics, t-tests and multiple linear regression were used to analyze the data.

**Results:**

A total of 156 trauma unit nurses participated in the study. The majority of the nurses are aged 31 to 40 years (81.4%), married (89.7%), and hold a Bachelor of Science in Nursing degree (87.2%). Female nurses report significantly lower burnout levels (23.0 ± 5.2) compared to male nurses (25.6 ± 5.4, *p* = 0.002). Nurses not wishing to continue in the trauma unit report higher burnout (30.1 ± 4.0) than those who do (23.5 ± 5.2, *p* = 0.001). Single nurses exhibit higher problem-focused coping scores (24.8 ± 5.0) compared to married nurses (22.0 ± 4.6, *p* = 0.023), and those in the Trauma Step Down Unit also show higher scores (24.9 ± 3.6), *p* = 0.026. Single) nurses use more emotion-focused coping strategies (33.7 ± 6.2) than married nurses (30.2 ± 6.0), *p* = 0.03), and those in the Trauma Step Down Unit report higher scores (34.7 ± 6.0), *p* = 0.004). Charge nurses/head nurses report lower avoidant coping scores (15.8 ± 3.3) compared to registered nurses (19.0 ± 4.7), *p* = 0.022).

**Conclusion:**

These findings highlight the impact of personal and professional factors on nurses’ coping strategies and professional quality of life. The study underscores the necessity of tailored interventions to support nurses’ coping mechanisms, emphasizing the enhancement of problem-focused strategies and reduction of avoidant behaviors to improve overall well-being and job satisfaction.

## Introduction

Trauma nursing encompasses the comprehensive care of trauma patients throughout their confinement, involving highly unpredictable and emotionally taxing situations [1, 2]. Experience-wise, caring for trauma patients proved more difficult when it comes to caring for younger patients or those who lost a limb or an organ due to an accident, since it greatly affects their whole self-esteem and demeanour. Despite going through the nurses’ struggles as well as stressors in their professional and social lives, they ought to be able to care for their patients with the highest quality of care they can render regardless of what they are personally going through and the length of their duty hours. Despite such challenges, trauma nurses are expected to provide high-quality care regardless of personal and professional difficulties [3]. In a similar study [1], stated that a highly stressful environment and frequent exposures to trauma patients can increase the nurses’ risk of developing burnout (BO), compassion fatigue (CF), and secondary traumatic stress (STS). In a recent study, it was shown that burnout (59.8%) and STS (50.7%) are common, and trauma nurses had poor levels of compassion satisfaction (21%) [4]. Despite these work-related stresses being innate in trauma critical care nursing, many studies have established the potential ruin it has on the physical, emotional, and psychological well-being of nurses, which can harm their job performance.

As cited in Marwa’s (2019) study, recurrent exposure to dramatic events and unexpected loss can give the caregiver a sense of CF and BO due to physical and mental exhaustion, hardship, and the craving for a moment of disconnection from the surroundings. Although they are commonly used interchangeably, CF and burnout have different meanings. The concept of CF comprises of burnout and STS [5].

CF was first coined by Joinson in 1992 as a unique type of BO specifically experienced by those giving care to trauma patients which inhibits their ability to nurture [6]. CF varies from BO.

since it is a direct consequence of one’s exposure to others’ trauma whereas burnout may develop without being exposed to others’ trauma. It results in the loss of one’s capacity to adequately care for their patients [1]. It is often described as the “cost of care” and is said to be a natural behavior or feeling developing from the knowledge of the traumatizing events a patient underwent. The feeling of CF arises from the desire to help or from helping the traumatized person and this feeling further escalates when the ability to help is limited or unavailable or when the person dies or is more badly inflicted [7]. Moral distress compounds the feelings of CF. The fear of reviving the stressful, traumatic experience as well as the frustration from being unable to act in line with one’s convictions are components of CF. Patient engagement, the nurse’s personal resources, and stress exposure all contribute to the ongoing and cumulative process of CF [3]. People suffering from CF are emotionally exhausted, anxious, sad, lack enjoyment at work, and report experiencing insomnia [8]. Unlike BO, CF, though may occur suddenly, can have a faster recovery rate when recognized [9]. A study done in the United States found a significant positive relationship between CS and the level of assistance received from coworkers [1]. However, nurses may decide to abruptly leave their unit or their profession due to overwhelming CF. Caregivers suffer CF when repeatedly required to empathize with distressed patients, which can significantly contribute to caregiver BO.

Like CF, burnout can threaten a caregiver’s capability to render effective care and maintain professional as well as personal therapeutic relationships [10]. BO is noted to develop slowly and is usually noted in a burdensome structural environment [11]. It is a process rather than a static state which begins progressively and builds up over time [8, 12], defined burnout as feelings of despair, detachment, and indifference to the work environment [13]. also defined burnout as a process that involves emotional fatigue, depersonalization, and diminished perception of personal achievement. It may develop from lingering distress at the workplace due to conflict between work requirements and employers’ demands. It encompasses physiologic responses as well as emotional responses. Due to the demands of delivering emotionally taxing and intense treatment, trauma nurses have a higher than usual vulnerability to burnout compared to nurses in other specialties [14]. BO also negatively affect job performance, morale, and self-efficacy [13]. It has been associated with nurses’ job dissatisfaction as well as patient dissatisfaction [9]. A recent study revealed that with a high prevalence of burnout (58%), there was also a significant amount of STS 38%) and moral distress (3.4 ± 1.4) [3]. The other element of compassion fatigue is STS which was defined by [12] as “the natural consequent behaviors and emotions resulting from knowing about a traumatizing event experienced by a significant other or the stress resulting from helping or wanting to help a traumatized or suffering person” [10]. stated that STS is an indirect experience resulting from a traumatic event wherein the symptoms in caregivers are related to the patient’s experience rather than their own. The caliber of patient care is contingent upon a myriad of elements, including the psychological state of the care providers. Stress and burnout can impede the potential for substantial and secure engagement with patients and their families, while the assimilation and internalization of overwhelming emotions might culminate in the manifestation of secondary trauma [15]. Given the nature of a healthcare worker’s duties and responsibilities, it is said to be an inevitable part of their response when dealing with trauma patients [12]. Having a history of personal trauma increases a healthcare worker’s risk and vulnerability of developing STS compared to those without previous history [10].

Many researchers supported the stress and burnout of trauma nurses in different levels of trauma care. With an emphasis on the subcategory of CF, which includes BO and STS, the Professional Quality of Life Tool (ProQOL) is primarily used to assess levels of satisfaction and weariness connected to compassion. The experience of distressing life events and the perception of social support display a notable correlation with instances of suicide attempts [16]. To deal with distressing situations, nurses may use a variety of coping mechanisms. The BRIEF-COPE assessment tool was utilized to determine the nurses’ coping strategies in this study.

No studies conducted in Qatar sought to identify how caring for trauma patients affects trauma nurses’ professional quality of life. Additionally, a substantial gap exists in the existing literature concerning the examination of CF, BO, STS, and the associated coping proficiencies among trauma nurses. This study aims to assess the professional quality of life and coping strategies of trauma nurses in Qatar using tools such as the Professional Quality of Life (ProQOL) and BRIEF-COPE assessments. By identifying triggers and proposing interventions, the study aims to enhance the well-being and performance of trauma nurses.

## Methodology

### Setting and design

Data on professional quality of life among trauma nurses working at the trauma care units of Hamad General Hospital, Qatar was gathered using a cross-sectional design.

## Sampling and sample size

The research focused on eligible nurses who had been working in the trauma unit for at least 6 months and possessed a Qatar Council for Healthcare Practitioner license (QCHP license). The eligible nurses were those working in the trauma resuscitation unit, trauma intensive care unit, trauma step-down unit, and trauma surgical ward. The total population of interest was 220 nurses.

To determine the appropriate sample size, a finite population correction formula was used, with a desired margin of error or precision level of 5% and a confidence interval of 95%. Considering an assumed proportion of quality of life to be 50% among the nurses, the calculated sample size was 141. Considering a 10% non-response rate, the final sample size was set at 155 nurses. Data was collected using a convenience sampling technique between February 1 to May 15, 2023. Nurses who were deployed to another facility during the study period were not directly involved in patient care, were under probation period, or were on leave for more than 30 days during the study period were excluded.

## Data collection tool

The questionnaire contains the sociodemographic variables which consist of 12 items and two tools the ProQOL and Brief-COPE. The first tool was the Professional Quality of Life Scale (ProQOL), which consists of 30 items with sub-scales for compassion satisfaction, burnout, and STS in which the two latter fall under compassion fatigue [5]. This tool was designed to measure Compassion fatigue and compassion satisfaction in healthcare professionals by imploring the positive and negative aspects of working with patients who experienced trauma. The tool scored a five-point Likert scale ranging from 1 to 5 i.e. Never [1], Rarely [2], Sometimes [3], Often [4], and Very Often [5]. The total score ranges from 5 to 150, with higher scores indicating higher levels of Compassion satisfaction, BO, and STS. The total score was further categorized into three; 22 or less as Low, 23–41 as Moderate, and 42 or more as High Level of Compassion Satisfaction, BO, and STS.

The Brief-COPE tool was utilized to assess the coping strategies of the participants. It consists of 28 questions under the following subdomains: problem-focused coping (8 items), emotion-focused Coping (12 items), and avoidant coping (8 items). The tool scored a four-point Likert scale ranging from 1 to 4 which includes: I haven’t been doing this at all [1], A little bit [2], A medium amount [3], and I’ve been doing this a lot [4]. The total score ranges from 4 to 112, Higher scores represented an increased use of each coping style (Carver, 1997).

## Ethical considerations

Ethical considerations were considered, and the study adhered to the guidelines and principles outlined in the “Declaration of Helsinki.” Approval was obtained from the IRB of Hamad Medical Research Center (MRC) with the number MRC-01-22-483.

## Data collection

Data collection involved sending the questionnaire to participants through a Microsoft Form link in their corporate emails. Participants were provided in the e-mail with an information sheet and were informed that their participation was voluntary and anonymous. To ensure a high response rate, weekly e-mail reminders were sent to participants to complete the survey.

### Statistical analysis

The collected data were analyzed using STATA 17. Categorical and continuous data values were expressed as frequency (percentage) and mean ± SD or median and inter-quartile range (IQR) as appropriate. Descriptive statistics were used to summarize the demographic characteristics and data related to the professional quality of life and coping strategies. Burnout and quality of life scores were calculated to take the sum of all the questions of respective questionnaires. Quantitative data were analyzed and compared using an Unpaired t-test and One way ANOVA. In addition, linear regression analyses were used to see the association between compassionate fatigue, compassionate satisfaction, coping strategies and the demographic variables. All P values presented were two-tailed, and P values < 0.05 were considered statistically significant.

## Results

A total of 156 trauma unit nurses participated in this study with most of the nurses were aged 31 to 40 years (81.4%), were married (89.7%), and held a Bachelor of Science in Nursing degree (87.2%). Almost 82% of the respondents reported having children. The average years of experience in trauma unit nursing was 6.7 ± 4.5 years. Regarding the work area, 64.7% of the nurses worked in the trauma intensive care unit; 84% reported exposure to traumatic events; 80.8% usually worked 12-hour shifts; and 91% expressed willingness to continue working in the trauma unit (Table 1). Table 2 displays the professional quality of life and coping strategy scores. The trauma nurses had an average Compassion satisfaction score of 37.8 ± 6.3 (range: 19–50), a burnout score of 24.0 ± 5.4 (range: 11–36), and a secondary traumatic stress score of 24.8 ± 6.9 (range: 13–50). The mean problem-focused coping score was 22.3 ± 4.7 (range: 10–32), indicating a tendency to address problems directly. The average emotion-focused coping score was 30.6 ± 6.0 (range: 13–48), while the avoidant coping score averaged 18.7 ± 4.7 (range: 9–36) (Table 2).

**Table 1:** Participants characteristics

Variables	Level	Value
N		156
Age	25-30yrs	8 (5.1%)
	31-35yrs	84 (53.8%)
	36-40yrs	43 (27.6%)
	41-45yrs	11 (7.1%)
	>=45yrs	10 (6.4%)
Gender	Male	68 (43.6%)
	Female	88 (56.4%)
Education	Diploma	10 (6.4%)
	BSN	136 (87.2%)
	Masters	9 (5.8%)
	Ph.D.	1 (0.6%)
Experience in trauma Unit, mean ± SD		6.7 ± 4.5
Designation	Charge Nurse	11 (7.1%)
	Head Nurse	1 (0.6%)
	Registered Nurse	144 (92.3%)
Marital Status	Single	16 (10.3%)
	Married	140 (89.7%)
Number of Children	0	28 (17.9%)
	1–2	101 (64.7%)
	3–5	27 (17.3%)
Your Current Area of Employment	Trauma Intensive Care Unit	101 (64.7%)
	Trauma Resuscitation Unit	16 (10.3%)
	Trauma Step Down Unit	24 (15.4%)
	Trauma Surgical Unit	15 (9.6%)
Exposure to Traumatic Events	No	25 (16.0%)
	Yes	131 (84.0%)
Usual Working Hours	12 h	126 (80.8%)
	8 h	30 (19.2%)
Hours worked per week	40 h	95 (60.9%)
	41–50 h	60 (38.5%)
	51–60 h	1 (0.6%)
Wish to continue working in a trauma center	No	14 (9.0%)
	Yes	142 (91.0%)

**Table 2:** Professional quality of life and coping strategies scores and levels of trauma nurses

Variables		Value
N		156
Compassion satisfaction, mean (SD)		37.8 ± 6.3
Burnout, mean (SD)		24.1 ± 5.4
Secondary Trauma Stress, mean (SD)		24.8 ± 6.9
Problem focused, mean (SD)		22.3 ± 4.7
Emotion coping, mean (SD)		30.6 ± 6.0
Avoidant coping, mean (SD)		18.7 ± 4.7

**Fig. 1:**
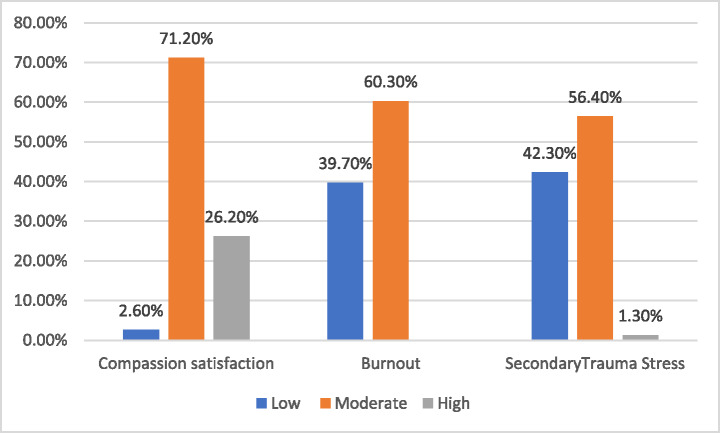
Levels of professional quality of life

Approximately 71% of trauma nurses reported a moderate level of compassion satisfaction, (*p* = 0.05), suggesting a potential but not definitive influence of age on perceived fulfillment. Only 2.6% reported low levels, suggesting a need for support and interventions. In the burnout domain, 60.3% of nurses had moderate levels of emotional exhaustion and depersonalization. For secondary traumatic stress, 56.4% had moderate levels, while only 1.3% reported high levels (Fig. 1).

Table 3 presents professional quality of life and coping strategies stratified by demographic and job-related variables.

**Table 3:** Association between coping strategies on professional quality of life dimensions and demographic factors

Variables	*N*	Professional Quality of life			Coping strategies		
		Compassion satisfaction	Burnout	Secondary trauma stress	Problem focused	Emotion coping	Avoidant coping
Age							
25-30yrs	8	38.8 ± 7.4	25.0 ± 5.8	22.9 ± 2.9	21.3 ± 3.5	27.0 ± 5.5	16.6 ± 5.6
31-35yrs	84	37.9 ± 5.8	24.3 ± 5.0	25.4 ± 6.7	22.4 ± 5.0	31.3 ± 6.4	19.6 ± 4.6
36-40yrs	43	36.0 ± 6.8	24.6 ± 6.3	24.2 ± 7.0	21.0 ± 4.2	29.1 ± 5.5	17.8 ± 3.9
41-45yrs	11	41.4 ± 6.9	24.0 ± 5.2	26.5 ± 10.8	25.0 ± 4.5	32.3 ± 5.7	19.8 ± 6.8
>=45yrs	10	40.8 ± 3.9	20.0 ± 3.6	22.8 ± 5.6	23.9 ± 3.5	32.4 ± 3.9	16.3 ± 3.7
p-value		0.05	0.17	0.55	0.07	0.08	0.05
*Gender*							
Male	68	37.1 ± 6.4	25.6 ± 5.4	25.8 ± 5.9	21.9 ± 4.9	30.8 ± 6.5	19.5 ± 4.3
Female	88	38.4 ± 6.2	23.0 ± 5.2	24.1 ± 7.6	22.5 ± 4.5	30.4 ± 5.7	18.1 ± 4.9
p-value		0.19	0.002	0.12	0.39	0.72	0.057
*Education level*							
Diploma	10	41.1 ± 3.0	20.3 ± 3.9	23.8 ± 6.1	23.3 ± 3.1	31.5 ± 3.4	17.6 ± 2.6
BSN	136	37.5 ± 6.4	24.4 ± 5.5	25.0 ± 7.1	22.1 ± 4.8	30.6 ± 6.2	18.8 ± 4.9
Masters/Ph.D	10	39.0 ± 6.6	24.5 ± 5.1	24.4 ± 5.9	23.4 ± 4.7	30.1 ± 6.3	18.4 ± 2.6
p-value		0.19	0.071	0.86	0.53	0.86	0.71
*Marital Status*							
Single	16	38.5 ± 7.9	24.9 ± 6.8	25.4 ± 8.3	24.8 ± 5.0	33.7 ± 6.2	20.1 ± 5.4
Married	140	37.8 ± 6.1	24.0 ± 5.3	24.8 ± 6.8	22.0 ± 4.6	30.2 ± 6.0	18.6 ± 4.6
p-value		0.66	0.52	0.72	0.023	0.03	0.21
*Number of children*							
No children	28	37.6 ± 7.7	25.4 ± 6.3	24.5 ± 7.1	22.8 ± 4.6	31.3 ± 6.3	19.0 ± 5.0
1–2	101	38.0 ± 5.8	24.0 ± 5.2	25.1 ± 7.0	22.5 ± 4.8	30.9 ± 6.1	19.1 ± 4.7
3–5	27	37.5 ± 6.7	23.1 ± 5.5	24.4 ± 6.7	20.9 ± 4.3	28.6 ± 5.4	17.0 ± 4.2
p-value		0.9	0.28	0.86	0.23	0.17	0.11
*Employment Area*							
Trauma Intensive Care Unit	101	38.3 ± 6.3	23.8 ± 5.4	25.0 ± 7.2	21.8 ± 4.6	30.0 ± 5.9	18.5 ± 4.9
Trauma Resuscitation Unit	16	37.4 ± 6.4	23.6 ± 6.4	24.7 ± 5.9	21.4 ± 5.3	29.6 ± 6.1	18.8 ± 4.3
Trauma Step Down Unit	24	37.5 ± 5.5	25.6 ± 3.2	25.5 ± 7.2	24.9 ± 3.6	34.7 ± 6.0	20.4 ± 4.7
Trauma Surgical Unit	15	35.7 ± 7.7	24.7 ± 7.1	23.1 ± 6.2	22.2 ± 4.7	29.5 ± 4.9	17.9 ± 2.8
p-value		0.49	0.46	0.77	0.026	0.004	0.29
*Exposure of traumatic events*							
No	25	37.2 ± 5.9	23.6 ± 5.2	24.9 ± 7.7	21.8 ± 4.5	31.5 ± 6.8	19.2 ± 5.4
Yes	131	38.0 ± 6.4	24.2 ± 5.5	24.8 ± 6.8	22.3 ± 4.7	30.4 ± 5.9	18.6 ± 4.6
p-value		0.58	0.63	0.98	0.57	0.41	0.56
*Working hours*							
12 h	126	37.9 ± 6.1	24.3 ± 5.6	24.6 ± 6.9	22.2 ± 4.4	30.6 ± 5.8	18.8 ± 4.6
8 h	30	37.6 ± 7.2	23.2 ± 4.8	25.7 ± 7.3	22.3 ± 5.7	30.6 ± 7.2	18.6 ± 5.2
p-value		0.81	0.31	0.45	0.98	0.97	0.83
*Continue to work in trauma unit*							
No	14	34.0 ± 7.7	30.1 ± 4.0	25.1 ± 7.4	22.4 ± 4.1	30.5 ± 5.5	20.5 ± 4.1
Yes	142	38.2 ± 6.0	23.5 ± 5.2	24.8 ± 6.9	22.2 ± 4.7	30.6 ± 6.1	18.6 ± 4.7
p-value		0.016	< 0.001	0.9	0.93	0.95	0.14
*Designation*							
Charge nurse/head nurse	12	39.9 ± 3.0	21.9 ± 2.9	24.0 ± 7.4	21.3 ± 4.2	29.0 ± 5.0	15.8 ± 3.3
RN	144	37.7 ± 6.5	24.3 ± 5.6	24.9 ± 6.9	22.3 ± 4.7	30.7 ± 6.1	19.0 ± 4.7
*p*-value		0.24	0.15	0.66	0.48	0.34	0.022

A marginally significant association was observed between age and compassion satisfaction (*p* = 0.05), indicating a potential influence of age on perceived fulfillment. Nurses intending to remain in the trauma unit reported higher compassion satisfaction (38.2 ± 6.0) compared to those who did not (34.0 ± 7.7, *p* = 0.016), indicating a possible relationship warranting further investigation.

In terms of burnout, female nurses reported significantly lower levels (23.0 ± 5.2) than male nurses (25.6 ± 5.4, *p* = 0.002). Nurses not planning to remain in the trauma unit had notably higher burnout scores (30.1 ± 4.0) compared to those who intended to stay (23.5 ± 5.2, *p* = 0.001).

For problem-focused coping, single nurses scored higher (24.8 ± 5.0) than married nurses (22.0 ± 4.6, *p* = 0.023). Nurses in the Trauma Step Down Unit also reported higher scores (24.9 ± 3.6, *p* = 0.026), indicating greater reliance on problem-solving approaches.

Emotion-focused coping was more common among single nurses (33.7 ± 6.2) compared to married nurses (30.2 ± 6.0, *p* = 0.03). Similarly, nurses in the Trauma Step Down Unit showed higher emotion-focused coping scores (34.7 ± 6.0, *p* = 0.004), suggesting greater emotional processing of stress.

Avoidant coping scores were significantly lower among charge/head nurses (15.8 ± 3.3) compared to registered nurses (19.0 ± 4.7, *p* = 0.022), indicating a preference for more proactive coping styles among those in leadership roles.

Table 4 summarizes regression results. Regression analyses indicated associations between coping strategies and professional quality of life outcomes; however, these findings do not establish causality due to the study design. Compassion satisfaction was positively associated with problem-focused coping (β = 0.64, 95% CI: 0.37 to 0.90, *p* < 0.001) and negatively associated with avoidant coping (β = −0.23, 95% CI: −0.46 to − 0.01, *p* = 0.043). Nurses intending to continue to remain in the trauma unit reported significantly higher compassion satisfaction (β = 3.84, 95% CI: 0.82 to 6.86, *p* = 0.013).

**Table 4:** Multiple linear regression exploring the influence of coping strategies on professional quality of life dimensions and demographic factors

Variables	β (95% CI)	*p* value
*Professional quality of life—compassion satisfaction		
Problem focused	0.64 (0.37,0.9)	< 0.001
Emotional coping	0.09 (−0.14,0.33)	0.423
Avoidant coping	−0.23 (−0.46, −0.01)	0.043
Continue to work in trauma unit		
No	ref	
Yes	3.84 (0.82,6.86)	0.013
**Professional quality of life—burnout		
Problem focused	−0.41 (−0.62, −0.19)	< 0.001
Emotional coping	−0.03 (−0.22,0.15)	0.729
Avoidant coping	0.55 (0.36,0.73)	0.000
Gender		
Male	ref	
Female	−1.06 (−2.51,0.39)	0.152
Continue to work in trauma unit		
No	ref	
Yes	−5.28 (−7.77, −2.79)	< 0.001
***Professional quality of life—secondary trauma stress		
Problem focused	−0.43 (−0.71, −0.15)	0.003
Emotional coping	0.25 (0,0.49)	0.051
Avoidant coping	0.75 (0.51,0.99)	< 0.001

*Model 1 Compassion satisfaction adjusted with problem focused, emotional coping, avoiding coping and nurses want to continue to work in same unit

**Model 2 Burnout adjusted with problem focused, emotional coping, avoiding coping, gender and nurses want to continue to work in same unit

***Model 3 secondary trauma stress was adjusted with problem focused, emotional coping, avoiding coping

In contrast, Burnout was negatively associated with problem-focused coping (β = −0.41, 95% CI: −0.62 to − 0.19, *p* < 0.001) and intention to stay in the trauma unit (β = −1.06, 95% CI: −2.51 to − 0.39, *p* < 0.001), while positively associated with avoidant coping (β = 0.55, 95% CI: 0.36 to 0.73, *p* < 0.001).

Secondary traumatic stress showed a negative association with problem-focused coping (β = −0.43, 95% CI: −0.71 to − 0.15, *p* = 0.003) and a positive association with avoidant coping (β = 0.75, 95% CI: 0.51 to 0.99, *p* < 0.001).

## Discussion

This research investigated burnout, secondary traumatic stress, and compassion satisfaction among trauma nurses, providing insights into the effectiveness of different coping measures in mitigating stress. It also explored the association between professional quality of life and coping strategies among trauma nurses. The study’s cross-sectional design prevents making causal inferences, and it was a single-center study conducted with a sample of 156 nurses. Additionally, information on the professional quality of life and coping levels was not available in other studies for this population.

The study found that trauma nurses generally experience low to average levels of CF while maintaining elevated levels of compassion satisfaction. High compassion satisfaction is inversely related to BO and CF, as supported by [1, 17]. Nurses with higher compassion satisfaction scores also had lower compassion fatigue and burnout scores, suggesting that deriving satisfaction from their work helps them cope with job-related challenges more effectively [1].

Age and gender were identified as factors influencing nurses’ professional quality of life. Contrary to [11], who found no significant relationship between age and compassion satisfaction, our study noted a marginally significant association (*p* = 0.05). Older nurses, particularly those over 40, reported higher compassion satisfaction, aligning with findings by [6, 18]. This may be due to the greater life and work experience of older nurses, which helps them maintain a positive outlook and reduces the risk of compassion fatigue.

Gender differences were also significant, with males experiencing higher burnout levels (*p* = 0.002) compared to females. This finding contrasts with [19] who reported that females are more likely to experience burnout. While our study did not directly assess work-life balance or emotional expression, it is possible that cultural or workplace factors may influence how male nurses cope with stress, potentially contributing to higher burnout levels. This hypothesis aligns with prior literature suggesting that men may be less likely to seek emotional support or express vulnerability [9]. Other studies did not find a significant relationship between gender and professional quality of life [11, 18].

Coping strategies varied among nurses. Single nurses used more problem-focused coping strategies (*p* = 0.023) and emotion-focused strategies (*p* = 0.03) than their married counterparts. Nurses in the Trauma Step Down Unit exhibited higher use of both problem-focused (*p* = 0.026) and emotion-focused coping (*p* = 0.004) compared to those in other units. These findings suggest that coping strategies are influenced by both personal and professional factors. These findings underscore the importance of considering both personal circumstances and job-specific factors when developing interventions to support nurses’ coping strategies. Tailored support programs that address the unique needs of different nurse demographics and unit-specific challenges could enhance overall well-being and professional quality of life. This aligns with previous research indicating that coping strategies are significantly shaped by individual and situational factors [1, 17]. Understanding these nuances can help healthcare administrators create more effective support systems for their nursing staff, ultimately improving job satisfaction and reducing BO and staff turnover.

Avoidant coping strategies were less common among charge nurses/head nurses compared to registered nurses (*p* = 0.022). Avoidant coping negatively impacts compassion satisfaction and increases stress levels, leading to higher BO and STS [20]. Problem-focused coping, on the other hand, is associated with lower BO and STS, enhancing compassion satisfaction [17].

Nurses employed a mix of coping techniques, including seeking emotional support, positive reframing, and planning. Emotion-focused coping involved seeking support from family and colleagues, while problem-focused coping included active coping and seeking informational support. Avoidant coping, such as self-distraction and denial, was less common but associated with higher stress and anxiety levels [1, 15, 21–23].

### Limitation

This study has several limitations. First, the study’s cross-sectional and single-center design limits the ability to infer causal relationships and generalize findings beyond the study setting. Second, as a single-center study conducted at Hamad General Hospital using a convenience sample, the findings may not be generalizable to other settings or regions. Third, self-reported data may be subject to recall and response biases, which could influence the accuracy of reported professional quality of life and coping measures. Additionally, important variables such as personality traits, organizational culture, social support, and specific workplace stressors (e.g., violence, moral distress) were not included, limiting the comprehensiveness of the analysis. The absence of baseline or longitudinal data restricts the ability to assess changes over time or the impact of interventions. Future studies employing longitudinal, multicenter, and mixed-method designs are needed to deepen understanding of coping dynamics and professional well-being among trauma nurses.

## Conclusions

This study identified moderate levels of burnout and secondary traumatic stress alongside relatively high compassion satisfaction among trauma nurses in a tertiary care hospital in the Gulf region. Certain coping strategies, particularly problem-focused coping, were associated with more favorable professional quality of life outcomes, while avoidant coping was linked to higher stress and burnout. Demographic and job-related differences in coping styles were also observed, suggesting the need for tailored support interventions. However, these findings should be interpreted with caution due to the cross-sectional design, single-center setting, and reliance on self-reported data, which limit the ability to draw causal inferences and generalize results. Further research using larger, more diverse samples and longitudinal methodologies is essential to better understand the complex relationships between coping mechanisms, workplace factors, and long-term well-being among trauma nurses.

## Data Availability

The availability of data and materials will be available on the subject request because of certain access restrictions, such as ethical, legal, or commercial sensitivities but will be available from the corresponding author on reasonable request.
